# The Effects of Acupuncture Stimulation for Brain Activation and Alcohol Abstinence Self-Efficacy: Functional MRI Study

**DOI:** 10.1155/2017/2850124

**Published:** 2017-02-09

**Authors:** Chae Ha Yang, Seong Hun Choi, Ju Sang Kim, Yeon Hee Ryu, Young Jin Lim, Moon Seup Kim, Jeong woo Sohn, Sung Suk Oh, Cheongtag Kim, Mi Young Lee

**Affiliations:** ^1^Department of Physiology, College of Oriental Medicine, Daegu Haany University, Daegu, Republic of Korea; ^2^Department of Anatomy and Histology, College of Oriental Medicine, Daegu Haany University, Daegu, Republic of Korea; ^3^Department of Medical Science, Graduate School, Daegu Haany University, Gyeongsan-si, Republic of Korea; ^4^KM Fundamental Research Division, Korea Institute of Oriental Medicine, Daejeon, Republic of Korea; ^5^Department of Psychology, College of Social Sciences, Daegu University, Gyeongsan-si, Republic of Korea; ^6^Department of Secondary Special Education, College of Biomedical Science, Daegu Haany University, Gyeongsan-si, Republic of Korea; ^7^Medical Device Development Center, Daegu-Gyeongbuk Medical Innovation Foundation, Daegu, Republic of Korea; ^8^Department of Psychology, College of Social Sciences, Seoul National University, Seoul, Republic of Korea; ^9^Department of Physical Therapy, College of Biomedical Science, Daegu Haany University, Gyeongsan-si, Republic of Korea

## Abstract

We attempted to investigate whether acupuncture stimulation at HT7 can have an effect on brain activation patterns and alcohol abstinence self-efficacy. Thirty-four right-handed healthy subjects were recruited for this study. They were randomly assigned into two groups: the HT7 (Shenmen) group and the LI5 (Yangxi) group. Acupuncture stimulation was performed using a block paradigm during fMRI scanning. Additionally, the Korean version of Alcohol Abstinence Self-Efficacy Scale (AASES) was used to determine the effect of acupuncture stimulation on self-efficacy to abstain from alcohol use. According to the result of fMRI group analysis, the activation induced by HT7 stimulation was found on the bilateral postcentral gyrus, inferior parietal lobule, inferior frontal gyrus, claustrum, insula, and anterior lobe of the cerebellum, as well as on the left posterior lobe of the cerebellum (*p* < 0.001, uncorrected). According to the AASES analysis, the interaction effect for gender and treatment was marginally significant (*F*(1, 30) = 4.152, *p* = 0.050). For female group, the simple main effect of treatment was significant (*F*(1, 11) = 8.040, *p* = 0.016), indicating that the mean change score was higher in the HT7 stimulation than in the LI5 stimulation. Therefore, our study has provided evidence to support that HT7 stimulation has a positive therapeutic effect on the alcohol-related diseases.

## 1. Introduction

Acupuncture is one of the most popular alternative and complementary therapies [1–9]. It has been widely used to treat various neuropsychiatric diseases, such as depression [5, 8], insomnia [7, 9], and anxiety [6]. In addition, it has also been applicable in treating substance-related diseases, such as alcohol, cocaine, and nicotine dependence [1–4]. Regarding the treatment effect for substance-related diseases, previous studies have demonstrated that acupuncture may be associated with regulating dopamine neurons, thereby inducing a reduction of craving and withdrawal symptoms [10, 11].

Recent neuroimaging techniques have helped better understand the neural mechanisms of acupuncture [12–18]. In particular, functional MRI has been widely applied in research associated with acupuncture due to its advantages of noninvasive and high spatial resolution [19]. There have been many neuroimaging studies that identified brain activation regions induced by acupuncture with various acupoint sites and stimulation type [12–16].

It is well-known that each acupoint has a different indication, so that it is applied in accordance with the therapeutic purpose [20]. It has been reported that, among many acupoints, acupuncture at HT7 (Shenmen) has considerable therapeutic effect on the neuropsychological impairments and enhances self-control for emotional stimuli [20, 21]. However, a few neuroimaging studies have elucidated the brain effect for acupuncture at HT7 in the human brain. In this study, we investigated whether acupuncture stimulation at HT7 can have an effect on brain activation pattern and alcohol abstinence self-efficacy.

## 2. Subjects and Methods

### 2.1. Subjects

Thirty-four right-handed healthy subjects (21 males; mean age 22.09 ± 1.26 years, range 20~24 years) with no history of neurological, physical, or psychiatric illness were recruited. In addition, subjects included drinker from light to heavy alcohol consumption. Subjects were excluded if they reported no experience of alcohol use in their lifetime. All subjects were required to be alcohol-free for at least one week prior to the experiment. We randomly assigned each subject to either the HT7 (Shenmen; experimental acupoint; 9 males, 8 females) group or the LI5 (Yangxi; control acupoint; 12 males, 5 females) group (Table 1). All subjects provided written, informed consent prior to participation. The study protocol was approved by the Institutional Review Board of Daegu Oriental Hospital of Daegu Haany University.

### 2.2. Functional MRI

#### 2.2.1. Experimental Paradigm

**Figure 1:**
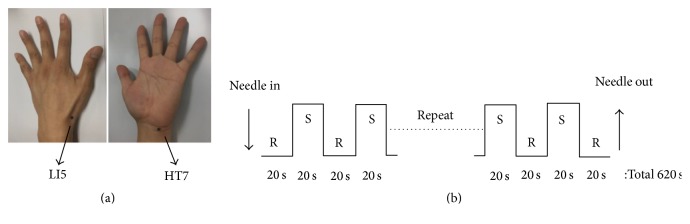
(a) Acupoint of HT7 (Shenmen: experimental acupoint) and LI5 (Yangxi: control acupoint). HT7 is located in the ulnar end of the crease of the wrist, in the depression of the radial side of the tendon of the flexor carpi ulnaris muscle of the wrist. LI5 is located on the radial side of the wrist in a depression between extensor pollicis longus and brevis tendons. (b) Experimental block design of fMRI. Acupuncture was applied at each acupoint, followed by 15 blocks of 20 s stimulation and 16 blocks of 20 s rest. S: stimulation. R: rest.

All subjects were examined in a supine position and firmly secured with an immobilizing frame. During fMRI scanning, acupuncture stimulation was performed using a block paradigm that consisted of 15 stimulation blocks alternating with 16 rest blocks. The duration of each block was 20 sec. Subjects in the HT7 group received needle acupuncture at the left HT7 (located on the ulnar end of the crease of the wrist, in the depression of the radial side of the tendon of the flexor carpi ulnaris tendon of the wrist), while subjects in the LI5 group received needle acupuncture at the left LI5 (located on the radial side of the wrist in a depression between extensor pollicis longus and brevis tendons) [22]. Acupuncture needle (nonmagnetic titanium sterile, 20 mm in length and 0.30 mm in diameter) was vertically inserted into each acupoint, with a depth of 0.5 cm, and it was kept in the acupoint. During the stimulation blocks, the needle was rotated manually clockwise and counterclockwise at 0.5 Hz. All procedures were performed by an experienced and licensed Korean medical doctor. The experimental block paradigm is shown in Figure 1.

The MAGNETOM Skyra 3T MRI System (Siemens, Erlangen, Germany) and the standard head coil at DGMIF (Daegu-Gyeongbuk Medical Innovation Foundation) were used to perform blood oxygenation level-dependent (BOLD) fMRI. BOLD-weighted Echo Planar Imaging (EPI) parameters were as follows: Repetition Time (TR)/ Echo Time (TE) = 2 sec/30 msec, field of view (FOV) =210 mm, flip angle = 90°, matrix size = 64 × 64, and slice thickness = 4 mm. For anatomical reference images, 28 axial, 4 mm slice thick, T1-weighted, spin echo images were obtained with a matrix size of 128 × 128 and FOV of 210 mm. Total images were acquired parallel to the bicommissural line of the anterior commissure-posterior commissure.

#### 2.2.2. fMRI Data Analysis

An analysis of the fMRI data was performed using statistical parametric mapping software (SPM 8: Wellcome Department of Cognitive Neurology, London, UK) implemented in the MATLAB environment (The MathWorks, USA). All images were realigned and normalized and, then, smoothed spatially with a Gaussian kernel at a full width at half maximum (FWHM) of 8 mm to improve the signal-to-noise ratio. The first level of analysis for each subject's contrast images was conducted to investigate the individual brain activation maps. The second level of analysis was performed using a random effect model with one-sample* t*-tests to examine the activation pattern of each acupuncture group. The images were then registered to the standard stereotaxic space of the Talairach coordinates to create statistical parametric maps that document the group average. Two-sample *t*-test was used to compare the activation differences between the two types of acupuncture. Activation was based on clusters larger than 15 contiguous voxels. It was significant at an uncorrected threshold of *p* < 0.001.

### 2.3. Alcohol Abstinence Self-Efficacy Scale (AASES) Questionnaire

The Korean version of AASES was used to determine the effect of acupuncture stimulation for self-efficacy to abstain from alcohol use before and after fMRI scanning [23, 24]. AASES assesses the self-efficacy of individuals to abstain from drinking in 20 typical drinking stimulations. Twenty items were rated on a 5-point Likert scale, ranging from “not at all” (0 point) to “extremely” (4 points for self-efficacy to abstain from alcohol) with a total score ranging from 0 to 80. A higher total score indicates higher self-efficacy level to abstain from alcohol use. Internal consistency (Cronbach's alpha) coefficients of the 20-item Korean version AASES was .91 [24].

## 3. Statistical Analysis

See comment in PubMed Commons below Statistical analysis was performed using SPSS version 14.0 (SPSS Inc., Chicago, IL). 2 × 2 (group × sex) analysis of variance (ANOVA) was conducted on the change scores that were computed by subtracting AASEA scores at the preacupuncture (base line) period from those at postacupuncture period. The significance level was set at .05.

## 4. Results

**Figure 2:**
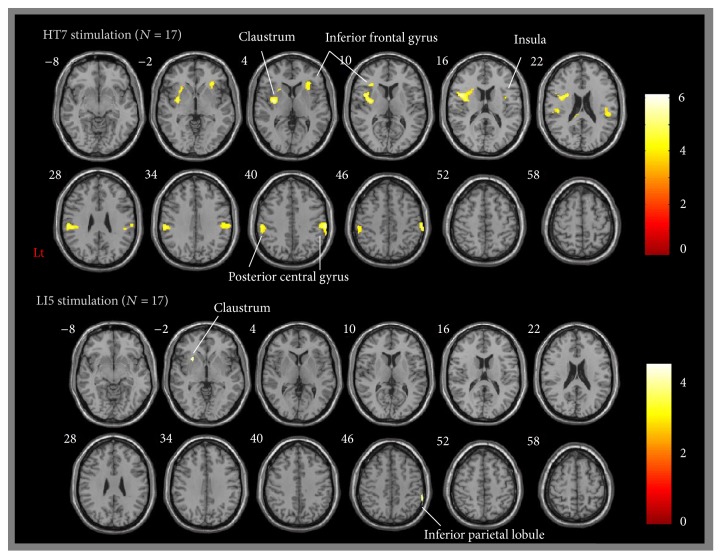
Brain activation with acupuncture at HT7 (above) and LI5 (bottom). In the HT7 stimulation, activated regions include bilateral postcentral gyrus, inferior parietal lobule, inferior frontal gyrus, claustrum, insula, and anterior lobe of the cerebellum, as well as left posterior lobe of the cerebellum. In LI5 stimulation, the activated regions include the right inferior parietal lobule and left claustrum. Statistical map was significant at *p* < 0.001 uncorrected level.

According to fMRI analysis, the activation induced by HT7 stimulation was observed on the bilateral postcentral gyrus, inferior parietal lobule, inferior frontal gyrus, claustrum, insula, and anterior lobe of the cerebellum, as well as on the left posterior lobe of the cerebellum. However, the activation induced by LI5 stimulation was only observed on the right inferior parietal lobule and left claustrum (Table 2, Figure 2).

Furthermore, HT7 stimulation as compared with the LI5 stimulation produced a higher BOLD signal response in the bilateral precuneus, precentral gyrus, postcentral gyrus, right middle occipital gyrus, posterior cingulate gyrus, medial frontal gyrus, cingulate gyrus, paracentral lobule, left superior temporal gyrus, and thalamus. However, no greater BOLD signal response was found by the LI5 stimulation compared with the HT7 stimulation (Table 3).

**Figure 3:**
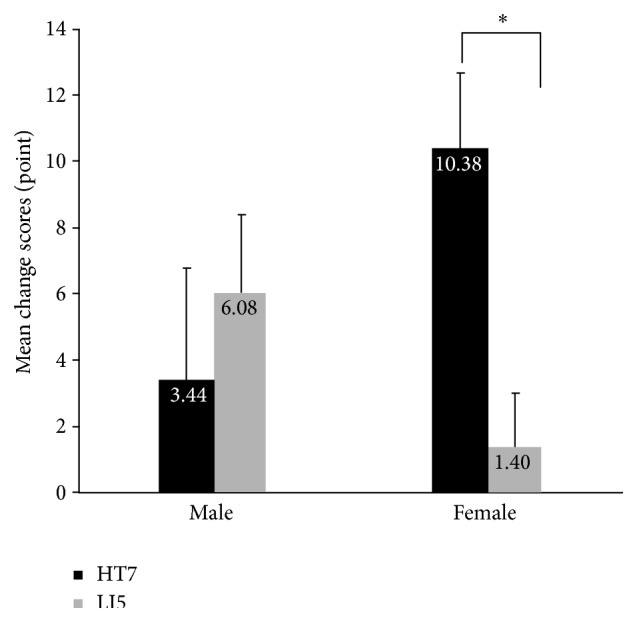
Comparison of AASEA score change between preacupuncture (base line) and postacupuncture period. For male group, the simple main effect of treatment was not significant. In contrast, for female group, the simple main effect of treatment was significant, indicating that the mean change score was higher in the H7 stimulation than in the LI5 stimulation. *∗* indicated *p* < 0.05.

As for the AASES analysis, two-way ANOVA was conducted to evaluate the effect of treatment (HT7 versus LI5) and gender on change scores from AASES. No main effect for treatment and gender was observed (*F*(1,30) = 0.115, *p* = 0.696; *F*(1,30) = 1.235, *p* = 0.275). An interaction effect for gender and treatment was marginally significant (*F*(1,30) = 4.152, *p* = 0.050). For male group, the simple main effect of treatment was not significant (*F*(1, 19)  =  0.443, *p*  = 0 .514). In contrast, for female group, the simple main effect of treatment was significant (*F*(1, 11)  = 8.040, *p* = 0.016), indicating that the mean change score was higher in the HT7 stimulation than in the LI5 stimulation (Figure 3).

## 5. Discussion

In the present study, we investigated whether acupuncture stimulation at HT7 can influence the brain activation pattern and alcohol abstinence self-efficacy. The main finding revealed that the bilateral postcentral gyrus, inferior parietal lobule, inferior frontal gyrus, claustrum, insula, and anterior lobe of cerebellum, as well as the left posterior lobe of the cerebellum, were activated when HT7 acupoint was stimulated. In contrast, LI5 control acupoint induced activation only on the right inferior parietal lobule and left claustrum. Alcohol abstinence self-efficacy level, on the other hand, indicated that mean change score was higher in the HT7 stimulation than in the LI5 stimulation for female group.

As previously reported, self-control for emotion occurs either by voluntary or by involuntary processing. Voluntary processing, such as conscious decision making, goal maintenance, and voluntary attention requires effortful control and involves brain areas, including the dorsolateral prefrontal cortex, intraparietal sulcus, and frontal eye field area. Moreover, involuntary processing such as salience detection, interoception, and conflict monitoring and resolution, and involuntary attention is automatic and involves the brain areas, including the bilateral insula cortex, anterior cingulate cortex, ventromedial prefrontal and orbitofrontal cortex, inferior frontal gyrus, and inferior parietal lobule [25–28]. Therefore, a dysfunction of the mentioned processing is one of the causes of substance-related diseases [29, 30]. In addition, many neuroimaging studies also reported alcohol-related diseases associated with several brain regions, including prefrontal cortex, thalamus, striatum, anterior cingulate cortex, and insula and others [31–35]. Our results suggest that HT7 stimulation could modulate brain activation patterns affected by alcohol-related diseases.

Previous neuroimaging studies have found some evidences for effect of HT7 stimulation [14, 17, 18]. Quah-Smith et al. (2010) reported that laser stimulation on the 4 acupoints (LR14, CV14, LR8, and HT7) to treat depression had resulted in an activation of frontal-limbic-striatal brain area in healthy subjects using fMRI [18]. Kang et al. (2013) also observed that HT7 stimulation had decreased smoking-related craving and activated brain regions involved in attention, motivation, and reward in male smokers [14]. In a recent study, Jung et al. (2015) discovered that acupoints (PC6 and HT7) stimulation induced brain activation in the posterior insula, posterior operculum, and the caudal part of the anterior cingulate cortex, enhancing bodily attention [17]. Similar to previous neuroimaging studies, we believe that our results can provide evidence regarding neural mechanism underlying the effect of HT7 stimulation.

AASES analyses provide some insights on gender differences in HT7 stimulation effects. The HT7 stimulation increases the alcohol abstinence self-efficacy for female but not for male. One explanation for this finding may relate to the differences in sensitivity of acupuncture between men and women. It is possible that even though HT7 made the similar physiological changes, women had high psychological reactivity to the changes enough to increase the self-efficacy level but men did not. Another possible explanation is that the experimental manipulation was not strong enough to change the self-efficacy level. Considering that the self-efficacy level cannot easily be changed with short-term stimulation, one-time acupuncture may not be sufficient to change the self-efficacy level.

In conclusion, we attempted to identify whether HT7 stimulation has a positive brain effect on the treatment of alcohol-related diseases. We found that brain areas activated by HT7 stimulation are associated with alcohol-related diseases. Based on our results, we suggest that HT7 stimulation will be applicable as an additional treatment for alcohol-related diseases. However, our study has some limitations that should be considered. First, the sample was drawn from a nonclinical population that did not have alcohol-related diseases. While nonclinical, only subjects that often drank alcohol were included in the study. Thus it is likely that this sample approximated the effect of HT simulation in clinical population. Second, even though subjects did not know which experimental groups (HT7 versus LI5) they belonged to, those who had knowledge of acupoint might have recognized which acupoints they were receiving. In that case, it may have caused a placebo effect. However, since no subject was majoring in oriental medicine, the possibility is low. Finally, we stimulated acupoint only one time. As such, our study was not sufficient to suggest psychological change. Therefore, further studies regarding the effect of repeated acupuncture treatment in individuals with alcohol-related diseases are necessary. Moreover, future studies should also examine the difference of neural mechanisms underlying acupuncture between genders.

## Figures and Tables

**Table 1:** Characteristics of subjects.

	HT7 group (*N* = 17)	LI5 group (*N* = 17)
Age (years )	21.94 ± 1.39	22.24 ± 1.15
Gender (F/M)	9/8	11/5
AUDIT	16.71 ± 6.10	15.53 ± 7.12
Frequency of drinking		
Monthly or less	8 (47.1%)	11 (64.7%)
2 to 4 times a month	6 (35.3%)	4 (23.5%)
2 to 3 times a week	3 (17.6%)	2 (11.8%)
Number of drinks per occasion		
1 or 2	2 (11.8%)	—
3 or 4	—	—
5 or 6	1 (5.9%)	5 (29.4%)
7, 8, or 9	6 (35.3%)	5 (29.4%)
10 or more	8 (47.1%)	7 (41.2%)

AUDIT: alcohol use disorder identification test.

**Table 2:** Significant brain activation area for acupuncture stimulation relative to rest block.

Brain region	Brodmann area	Peak MNI coordinates			Peak *t*-value	Number of voxels
		*x*	*y*	*z*		
HT 7 group: acupuncture stimulation-rest contrast						
Right						
Postcentral gyrus	1	62	−24	42	5.82	496
Inferior parietal lobule	40	60	−24	32	4.68	c
Inferior frontal gyrus	47	30	30	0	5.39	160
Claustrum	—	28	18	2	4.53	c
Cerebellum^a^	—	0	−54	−28	5.36	85
Insula	13	38	2	14	4.22	18
Left						
Claustrum	—	−36	−4	4	6.14	760
Insula	13	−44	6	12	4.22	c
Postcentral gyrus	1	−58	−26	38	4.82	481
Inferior parietal lobule	40	−58	−24	28	4.81	c
Cerebellum^b^	—	−18	−72	−36	4.78	29
Cerebellum^a^	—	−24	−48	−28	4.61	116
Inferior frontal gyrus	13	−36	26	10	4.40	30

LI5 group: acupuncture stimulation-rest contrast						
Right						
Inferior parietal lobule	40	60	−44	46	4.14	34
Left						
Claustrum	—	−28	16	−2	4.57	19

*p* value is uncorrected at *p* < 0.001. a: anterior lobe; b: posterior lobe; c: an activation area that belongs to the cluster listed in above.

HT7: experimental acupoint. LI5: control acupoint.

**Table 3:** Significant brain activation difference between HT7 and LI5 stimulation.

Brain region	Brodmann area	Peak MNI coordinates			Peak *t*-value	Number of voxels
		*x*	*y*	*z*		
HT7 group > LI5 group						
Right						
Precuneus	7	22	−58	36	4.00	59
Precentral gyrus	6	42	−14	28	3.86	40
Middle occipital gyrus	19	34	−74	18	3.85	177
Posterior cingulate gyrus	30	20	−52	18	3.84	98
Medial frontal gyrus	6	2	−6	48	3.72	51
Cingulate gyrus	24	10	−8	40	3.32	a
Paracentral lobule	5	20	−42	52	3.64	20
Postcentral gyrus	3	40	−22	46	3.56	30
Left						
Precentral gyrus	6	−42	−10	25	4.37	227
Precuneus	31	−20	−62	24	4.17	107
Superior temporal gyrus	38	−46	8	−8	4.05	34
Thalamus	—	−10	−7	4	3.79	477
Postcentral gyrus	1	−48	−22	54	3.64	30

LI5 group > HT 7 group						
NS	—	—	—	—	—	—

*p* value is uncorrected at *p* < 0.001. NS: nonsignificant.

a: an activation area that belongs to the cluster listed above.

HT7: experimental acupoint. LI5: control acupoint.
